# Repeat Breast-Conserving Surgery Versus Salvage Mastectomy for Ipsilateral Breast Tumour Recurrence After Breast-Conserving Surgery in Breast Cancer Patients: A Meta-Analysis

**DOI:** 10.3389/fonc.2021.734719

**Published:** 2021-11-23

**Authors:** Caiqin Mo, Weihong Ruan, Junyu Lin, Huaying Chen, Xiangjin Chen

**Affiliations:** ^1^ Department of Breast and Thyroid Surgery, The First Affiliated Hospital of Fujian Medical University, Fujian, China; ^2^ Department of General Surgery, Traditional Chinese Hospital of Xiamen, Fujian, China; ^3^ The First Clinical Medical College, Fujian Medical University, Fujian, China

**Keywords:** meta-analysis, repeat breast-conserving surgery, salvage mastectomy, ipsilateral breast tumor recurrence, breast cancer

## Abstract

**Background:**

Salvage mastectomy (SM) is the standard surgery for ipsilateral breast tumour recurrence (IBTR). However, whether repeat breast-conserving surgery (RBCS) is an alternative method remains unclear. We performed a meta-analysis to compare the effects of RBCS and SM after IBTR for breast-conserving surgery (BCS).

**Methods:**

We searched PubMed, Cochrane, Wiley Online and Embase for controlled studies comparing RBCS and SM after IBTR for BCS (published between 1993 and 2019, published in English). Our main endpoints were the secondary local recurrence rate (SLRR), distant metastasis rate (DMR) and overall survival (OS). We used a random-effects model or fixed-effects model for data pooling.

**Results:**

Fifteen of the 424 eligible studies were ultimately included, and all studies were retrospective cohort studies (n=2532 participants). 1) SLRR: The SLRR of RBCS was higher than SM (pooled relative rate (pRR) = 1.87, 95% CI 1.22 - 2.86, P=0.004). Stratified analysis was performed according to whether radiotherapy was performed after salvage surgery (radiotherapy group: 2ndRT, no radiotherapy group: no-2ndRT), and the following results were revealed: pRR=0.43 (95% CI 0.20-0.95, P=0.04) for group 2ndRT; and pRR=2.30 (95% CI 1.72-3.06, P<0.00001) for group no-2ndRT. These results showed that the main cause of heterogeneity was salvage radiotherapy. 2) DMR: No significant difference in the DMR was observed between RBCS and SM (pRR = 0.61, 95% CI 0.37 - 1.01, P=0.05). 3) OS: No significant difference in OS was observed between RBCS and SM (pRR=0.65, 95% CI 0.39 - 1.08, P=0.10).

**Conclusions:**

The SLRR of RBCS was higher than SM for ITBR after BCS, but survival was not affected. RBCS may be used as an alternative for IBTR patients after BCS with strict control for several indications, such as tumor size, recurrence interval and biological behavior, and attaching importance to subsequent salvage radiotherapy and systematic therapy.

## Introduction

Breast-conserving surgery (BCS) is a standard surgical method for early breast cancer. However, local recurrence exists. Ipsilateral breast tumor recurrence (IBTR) is defined as the reappearance of breast cancer in the region of the ipsilateral breast/chest wall or the draining regional lymph node basins (1). The 10-year ITBR rate is approximately 5-10% (2). For IBTR, approximately 6-7% of all patients have inoperable disease (3, 4), and 5-10% develop distant metastasis simultaneously (5, 6).

Opportunities for the detection of small and isolated IBTR diagnoses have increased (7), and the demands of repeat breast-conserving surgery (RBCS) have become more urgent. However, salvage mastectomy (SM) is the standard surgical method for operable IBTR (2, 8, 9), and whether RBCS is an alternative method in patients with IBTR is controversial. Some studies reported that the prognosis of RBCS was worse than SM (4, 10–12), but other studies have not (13–19). Therefore, we performed a meta-analysis of the secondary local recurrence rate (SLRR), distant metastasis rate (DMR) and overall survival (OS) of RBCS or SM in IBTR patients after BCS to further evaluate the feasibility of RBCS for IBTR after BCS.

## Materials and Methods

### Search Strategy

This meta-analysis is reported according to the Preferred Reporting Items for Systematic Reviews and Meta-Analyses (PRISMA).We selected 15 relevant studies published between 1988 and 2019 after searching Embase, PubMed, Cochrane, and Wiley Online databases (only published in English). We also searched the reference lists of important articles manually. The complete search strategy of PubMed is shown in **Data S1**.

### Selection and Data Extraction

The inclusion criteria were as follows: (a) studies comparing RBCS and SM of IBTR after BCS, regardless of whether radiotherapy was administered after the first BCS; (b) retrospective cohort studies or prospective cohort studies; and (c) studies that included data on the SLRR, DMR or OS.

The exclusion criteria were as follows: (a) unreasonable research design, incomplete data, or unclear endpoints; (b) poor data sources or sources from the same center; (c) data from the SEER database; and (d) studies not published in English.

### Quality Assessment

Two independent investigators reviewed the study titles and abstracts independently and extracted and analyzed the data, and disagreements were resolved by a third investigator. We extracted the following data: sample size, inclusion time, follow-up time, number/rate of secondary recurrence events, number/rate of distant metastatic events, number/rate of time of death, radiotherapy information and other treatment information. The risk for bias according to the PRISMA recommendations were assessed by two independent reviewers.

The Newcastle-Ottawa Scale (NOS) was used to assess the quality of the included literature. A higher score indicated a lower risk of bias. An overall NOS score ≥6 was considered acceptable (**Data S1**).

### Statistics Analyses

The following outcomes were assessed: SLRR, DMR and OS. We analyzed the SLRR, DMR and OS as binary count variables. If the 5-year and 10-year OS were provided, the 10-year OS was used as the final data. The overall relative risk (RR) was calculated and the Cochran Q test was used to assess heterogeneity between studies. I² testing was performed to assess the magnitude of the heterogeneity between studies, and if values was greater than 50%, moderate-to-high heterogeneity was indicated. A fixed-effect model was used for low heterogeneity, and a random-effect model was used for moderate-to-high heterogeneity. To confirm the effect of radiotherapy after RBCS on recurrence and survival, stratified analysis was performed for the SLRR based on whether radiotherapy was administered after RBCS.

We assessed the possibility of publication bias by funnel plots. We assessed funnel plot asymmetry by using Begg’s and Egger’s tests and defined significant publication bias as two-tailed p < 0.05. Sensitivity analysis was performed by omitting one study. We used Stata (version 12.0) and Endnote X9 for all statistical analyses.

## Results

### Study Selection Results

**Figure 1:**
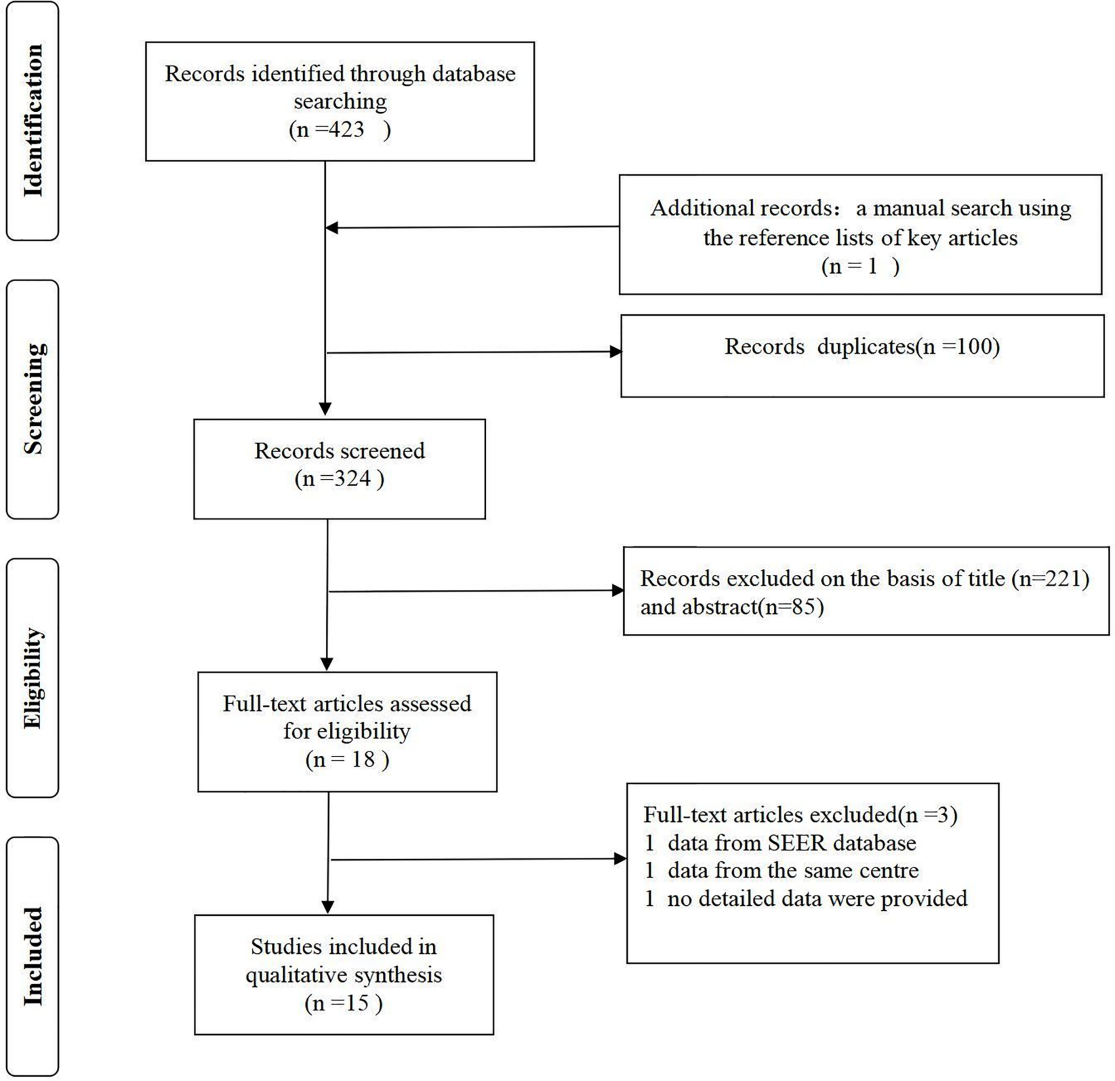
Flow chart of study selection.

A total of 424 studies were identified. No prospective randomized controlled studies were included. Fifteen studies (with 2532 participants) were ultimately included in our meta-analysis (**Figure 1**). The lowest NOS score of included studies is 7 and the detailed NOS study equality evaluation *via* the Newcastle-Ottawa Scale was shown in the supplementary materials **Data S1**.

### Study Characteristics

All studies were retrospective cohorts published between 1988 and 2019. The median follow-up time ranged from 52 months to 20 years. Of the 2532 participants, 633 underwent RBCS, and 1899 underwent SM. Our main endpoints were the SLRR, DMR and OS. Thirteen studies reported the SLRR, 4 studies reported the DMR, and 7 studies reported OS. Systematic data from the studies by Alpert, T.E. et al., Chen, S.L. et al. and Mccready et al. were not available, and the other 12 studies received certain systematic treatment. See **Table 1** for the detailed characteristics of the included studies.

**Table 1:** Characteristics of included studies.

Author	Published year	Country	Included time	Radiotherapy after primary breast-conserving	Radiotherapy after repeated breast-conserving	Endpoint	Systematic treatment	Median follow-up time	N
								RBCS SM	RBCS SM
Abner, A.L. (20)	1993	US	1968-1985	Yes	No	SLRR	Yes	79 (5-233) months	16 123
Alpert, T.E (13).	2005	US	Before1999	Yes	No	SLRR, DMR, OS	unclear	13.8 years	30 116
Chen, S.L (10).	2008	US	1988-2004	Yes	No	OS	unclear	10 years	179 567
Dalberg, K. (8)	1998	Switzerland	1976-1985	Yes, 79%	No	SLRR	Yes	13 (9-19) years	14 65
Fodor, J (14).	2008	Hungary	1983-1987	Yes, 50%	Yes, only 4	SLRR, OS	Yes	165 (75-240) months	32 32
Komoike, Y (15).	2005	Japan	1986-1993	Yes	No	SLRR, DMR, OS	Yes	20 years	55 88
Kurtz, J, M (21)	1988	Switzerland	before1985	Yes	No	SLRR	Yes	7 (1-20) years	52 66
Kurtz, J, M (22)	1990	French	1963-1982	Yes	No	SLRR、DMR	Yes	11 (5-24) years	34 36
Lee.J.H (23)	2015	Korea	1955-2011	Yes	Yes, partial	OS	Yes	NS	23 108
Mccready (24)	1994	Canada	1977-1986	No	Yes	SLRR	unclear	6 years	19 33
Salvadori, B (4).	1999	Italy	1970-1989	Yes	unclear	SLRR, DMR, OS	Yes	73 (1-192) months	57 133
Sellam (25)	2019	Israel	1987-2014	Yes	Yes, only 3	SLRR, OS	Yes	14 (1-30) years	47 74
Smanykó, V (26).	2019	Switzerland	1999-2015	Yes	Yes	SLRR, OS	Yes	59 (1-189)months 56 (3-189)months	39 156
Voogd, A.C (27)	1999	Netherlands	1980-1992	Yes	No	SLRR	Yes	52 months	20 229
Wapnir, I.L (12)	2017	Switzerland	2003-2010	Yes	Yes, only 3	SLRR	Yes	4.9 years	16 73

SLRR, secondary local recurrence rate; DMR, distant metastasis rate; OS, overall survival; RBCS, repeated breast-conserving surgery; SM, salvage mastectomy.

### Meta-Analysis Results

#### 1. SLRR

**Figure 2:**
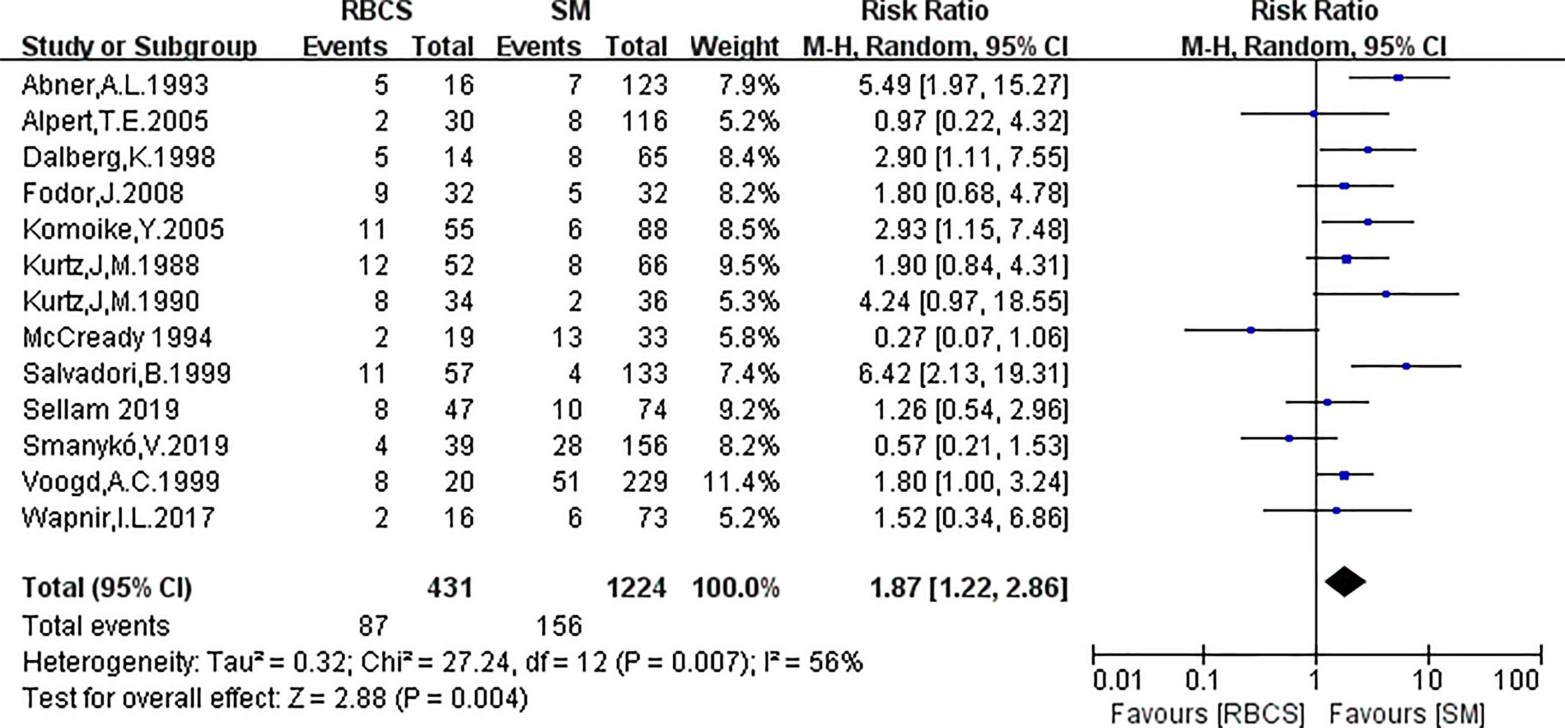
Forest plot of repeated breast-conserving surgery (RBCS) *versus* salvage mastectomy (SM) after ipsilateral breast tumor recurrence (IBTR), comparing secondary local recurrence rate (SLRR). The meta-analysis was performed with random effects model. RR more than 1 means the results favor SM group.

**Figure 3:**
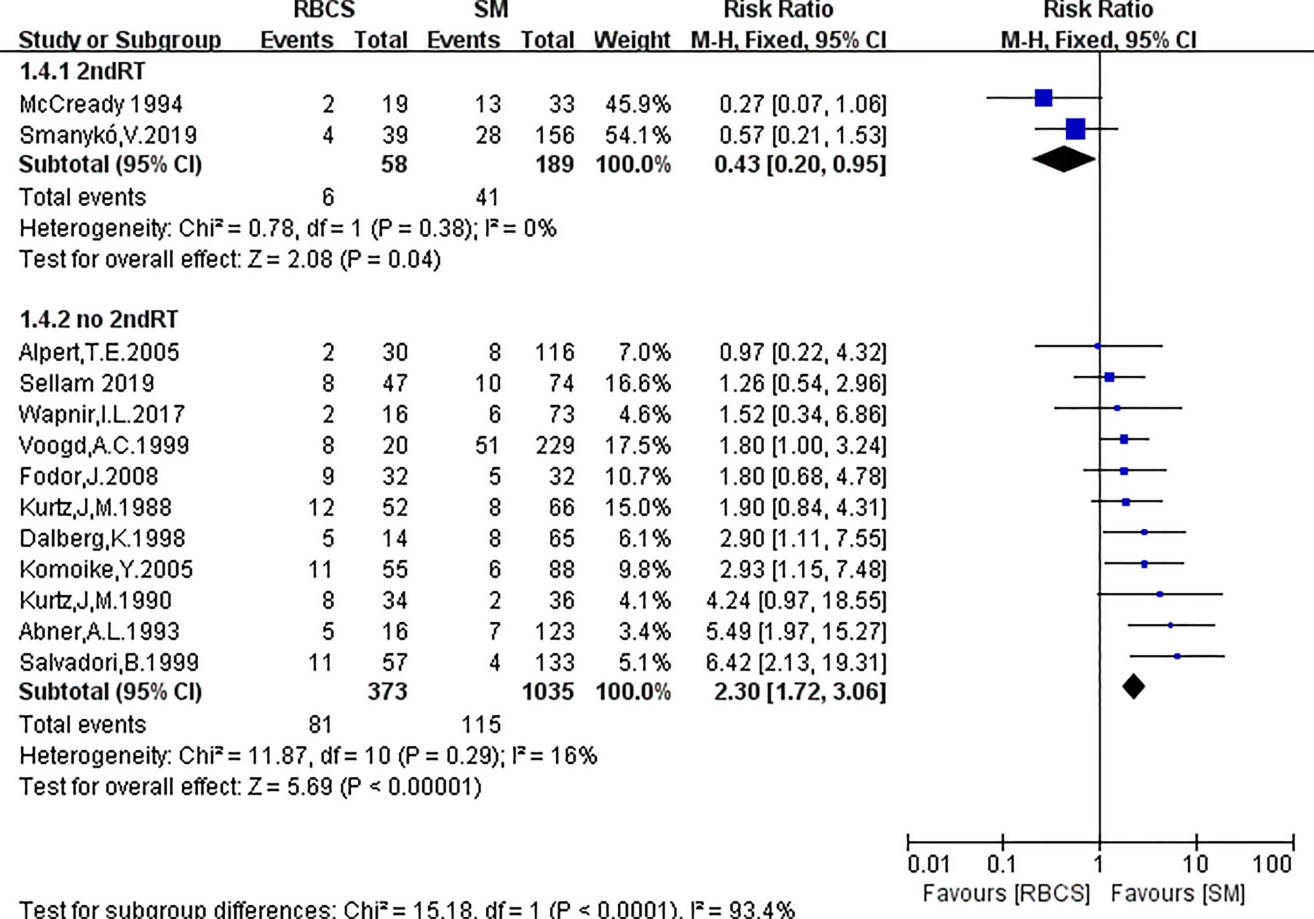
Forest plot of stratification analysis of repeated breast-conserving surgery (RBCS) *versus* salvage mastectomy (SM) after ipsilateral breast tumor recurrence (IBTR), comparing secondary local recurrence rate (SLRR). The meta-analyse was performed with fixed-effects model. RR more than 1 means the results favor SM group. 2ndRT: radiotherapy was performed after salvage surgery; no-2ndRT: radiotherapy was not performed after salvage surgery.

**Figure 4:**
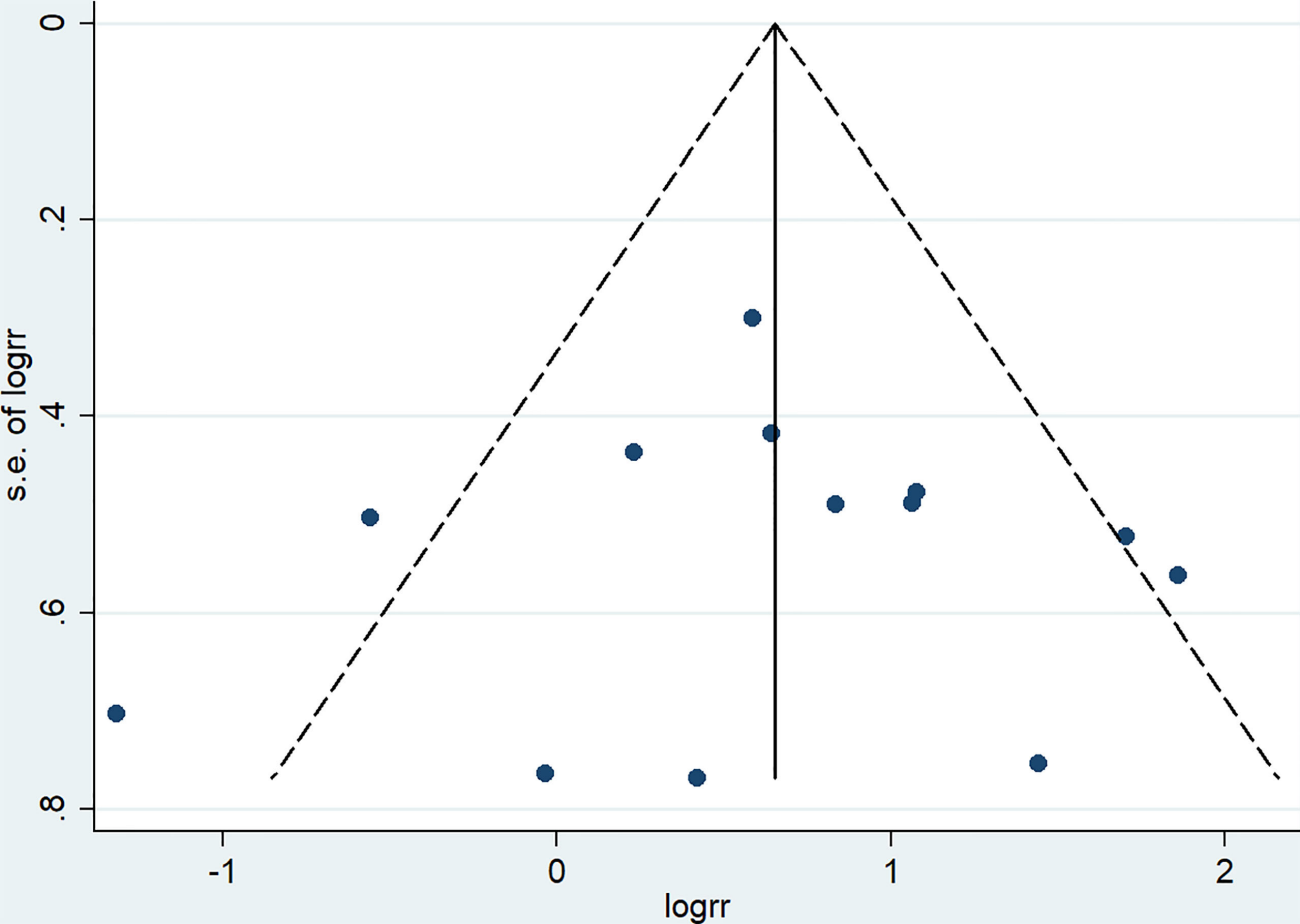
Funnel plot of studies included in meta-analysis of secondary local recurrence rate (SLRR).

**Figure 5:**
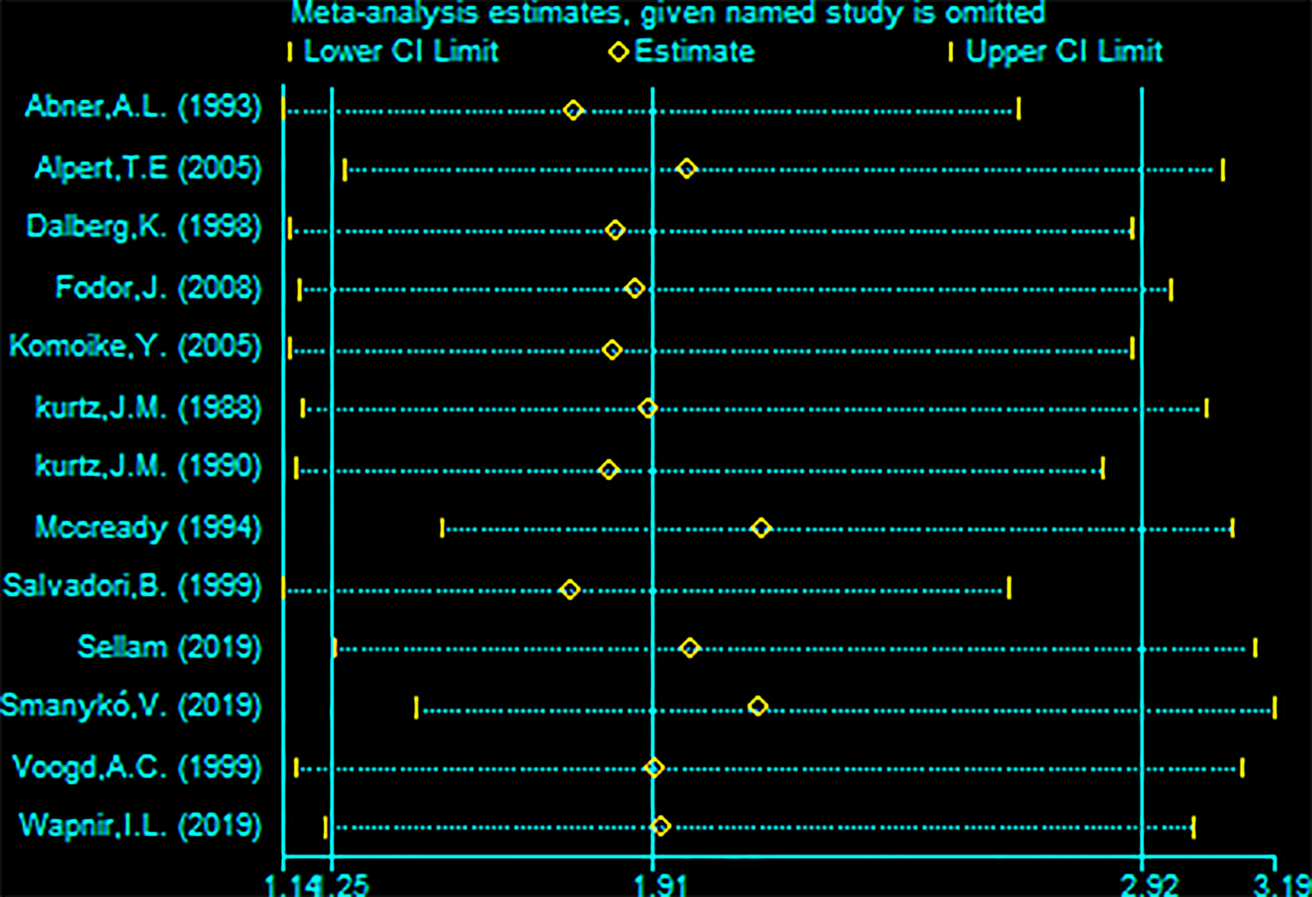
Sensitivity analysis of meta-analysis of secondary local recurrence rate (SLRR) by the method omitting one study.

A total of 13 studies reported the SLRR. Among these studies, 431 patients underwent RBCS, and 1224 patients underwent SM (I²=56%, P=0.007), which suggests heterogeneity among the studies. A random-effects model was used, and the combined effect size of pRR =1.87(1.22-2.86), P=0.004 (**Figure 2**) suggested that the SLRR of RBCS was significantly higher than SM. Stratified analysis was performed according to whether radiotherapy was performed after salvage surgery to examine sources of heterogeneity (**Figure 3**). The results revealed that radiotherapy after RBCS (2ndRT group) was performed in 2 studies, including 58 patients in the RBCS group and 189 patients in the SM group. Eleven studies did not perform radiotherapy after surgery (no-2ndRT), including 373 patients in the RBCS group and 1035 in the SM group. After stratification, there was no significant heterogeneity in the two groups (for 2ndRT, I²=0%,P=0.38, and for no-2ndRT, I²=16%, P=0.29). A fixed-effect model was used for stratification, and the results revealed a pRR of 0.43 for 2ndRT (95% CI 0.20-0.95, P=0.04) and a pRR of 2.30 for no-2ndRT (95% 1.72-3.06, P < 0.00001) (See the funnel plot in **Figure 4**). The results of Begg’s test (P=0.951)and Egger’s test (P=0.823) suggested no publication bias. Sensitivity analysis was performed by omitting one study (**Figure 5**). Removal of either study showed no significant effect on pRR.

#### 2. DMR

**Figure 6:**
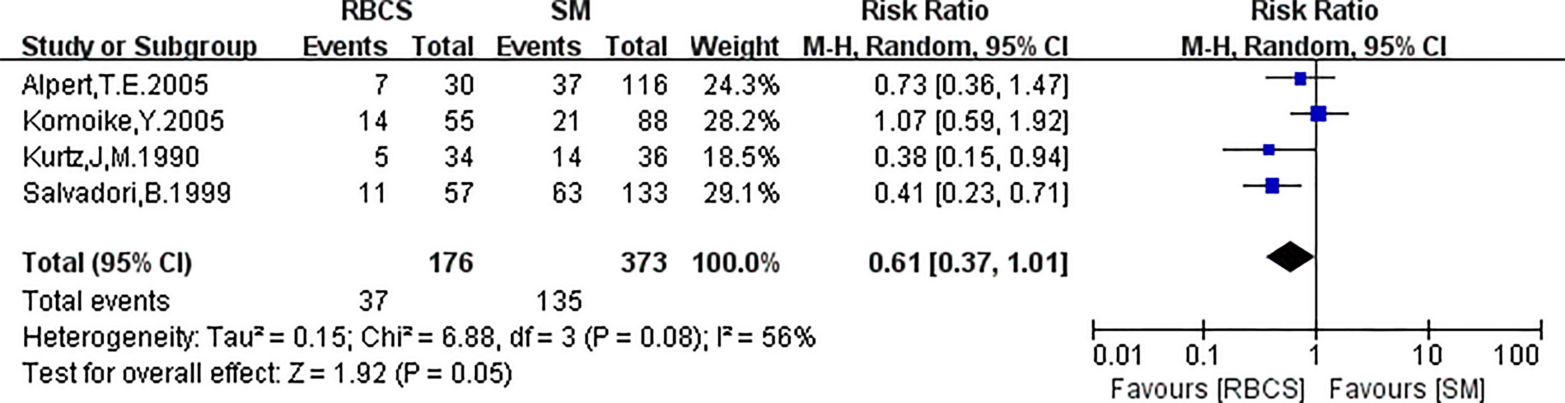
Forest plot of repeated breast-conserving surgery (RBCS) *versus* salvage mastectomy (SM) after ipsilateral breast tumor recurrence (IBTR), comparing distant metastasis rate (DMR). The meta-analyse was performed with random effects model. RR more than 1 means the results favor SM group.

**Figure 7:**
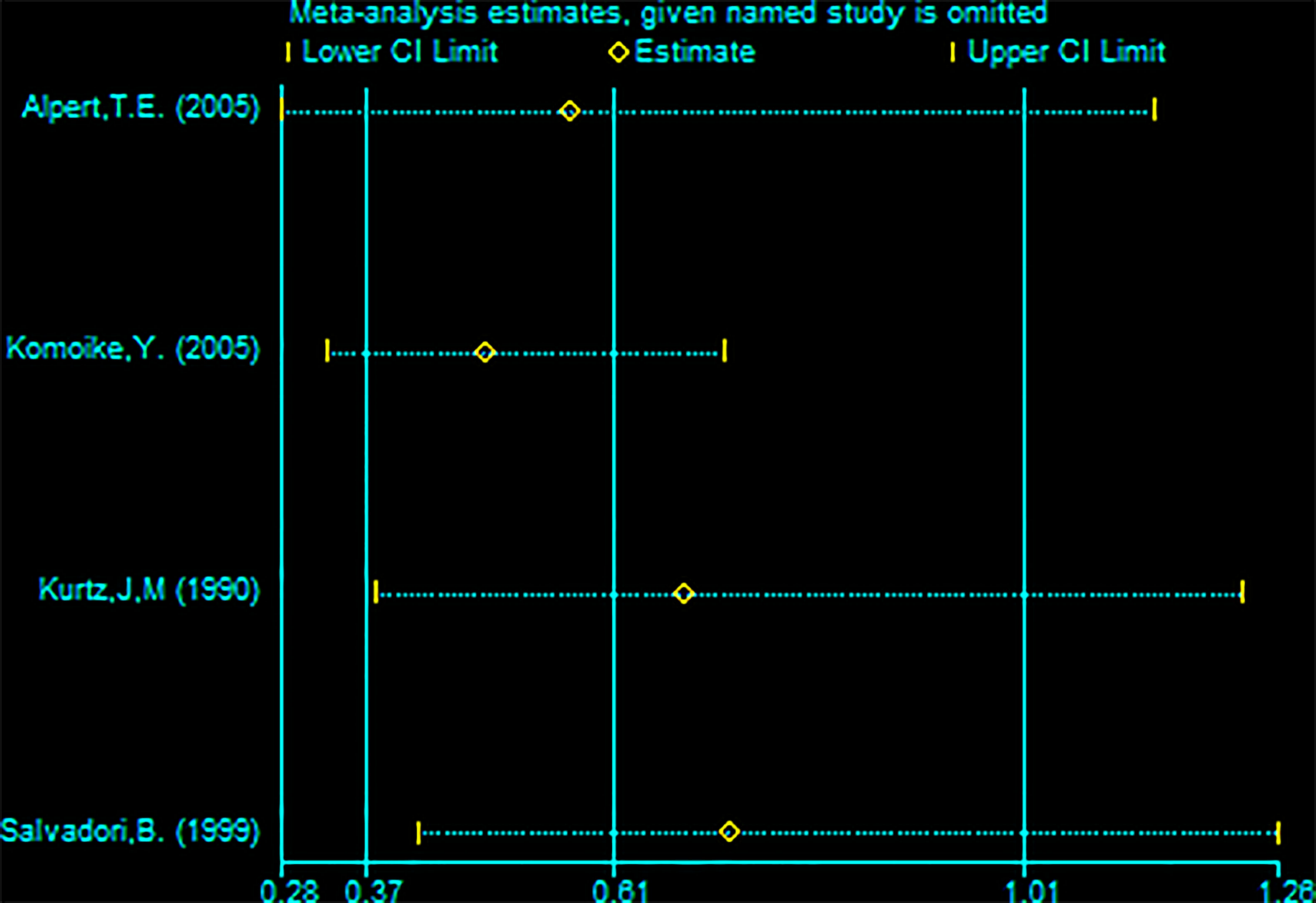
Sensitivity analysis of meta-analysis of distant metastasis rate (DMR) by the method omitting one study.

Four studies (with 176 RBCS and 373 SM participants) were included in the DMR analysis. The results suggested heterogeneity among the studies (I²=56%,P=0.08), and we used a random-effects model. The combined effect size [pRR=0.61,95% 0.37-1.01, P=0.05 (**Figure 6**)] showed no significant difference between RBCS and SM in DMR. The results of Begg’s test (P=1.0) and Egger’s test (P=0.747) showed no publication bias. Sensitivity analysis was performed using the method of omitting one study (**Figure 7**), and no significant changes in pRR were observed.

#### 3. OS

**Figure 8:**
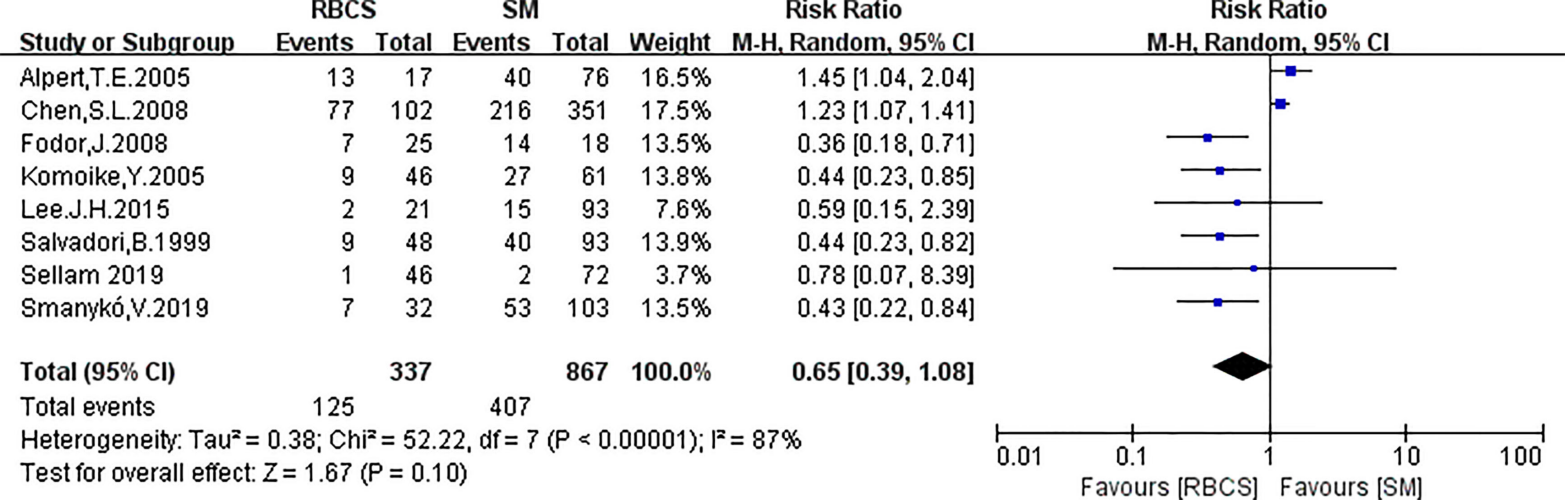
Forest plot of repeated breast-conserving surgery (RBCS) *versus* salvage mastectomy (SM) after ipsilateral breast tumor recurrence (IBTR), comparing overall survival (OS). The meta-analyse was performed with random effects model. RR more than 1 means the results favor SM group.

**Figure 9:**
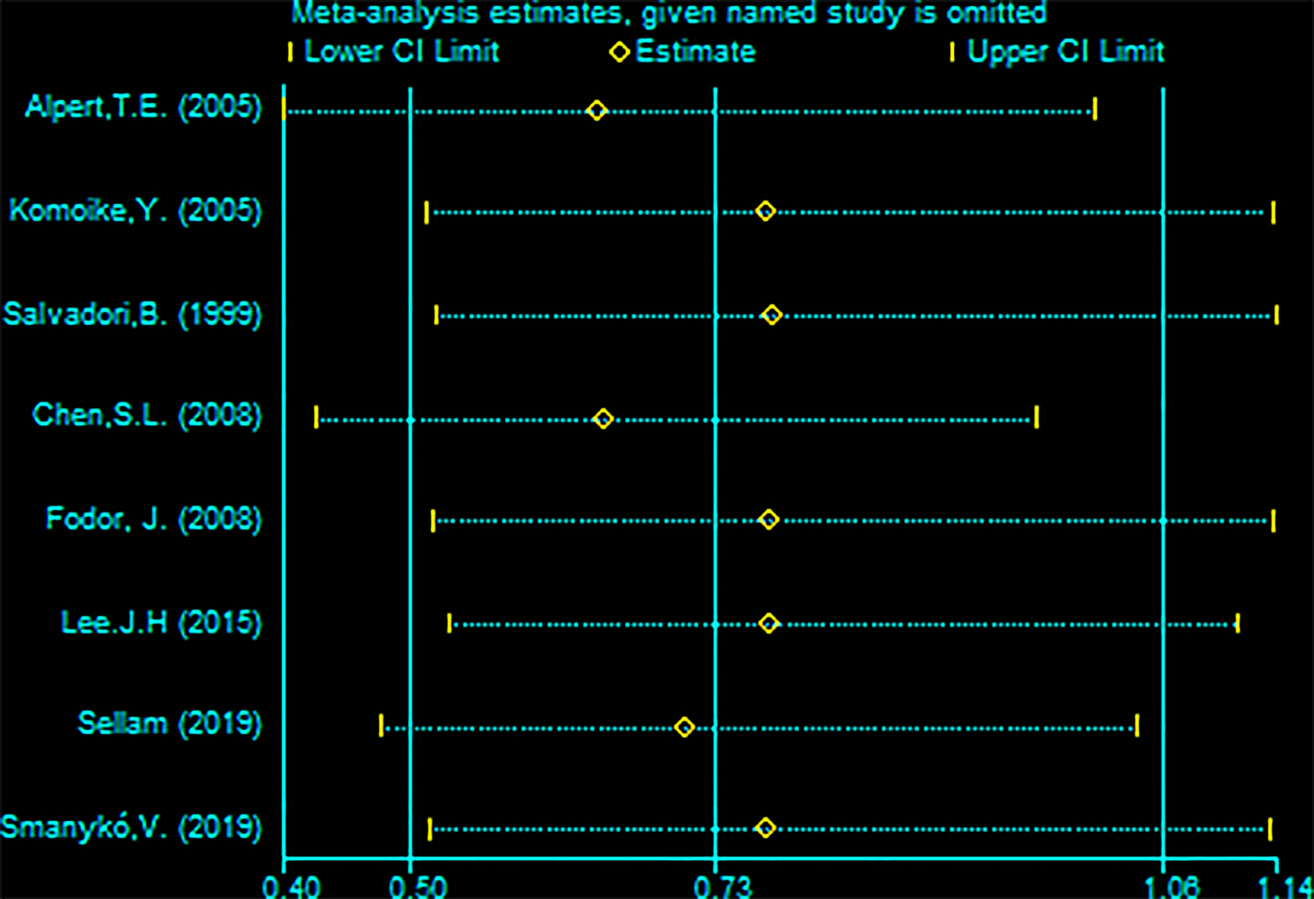
Sensitivity analysis of meta-analysis of overall survival (OS) by the method omitting one study.

Eight studies (with RBCS 337 and 867 participants) were included in the analysis of OS. The results suggested heterogeneity among the studies (I²=87%, P<0.00001), and we used a random-effects model. The combined effect size [pRR=0.65, 95% 0.39-1.08, P=0.10 (**Figure 8**)] showed no significant difference between RBCS and SM in OS. The results of Begg’s test (P=1.0) and Egger’s test (P=0.069) showed no publication bias. Sensitivity analysis was performed by omitting one study (**Figure 9**), and no significant changes in pRR were observed.

## Discussion

Since there is still some risk of recurrence after BCS, these patients face decisions of whether to undergo RBPS. For patients who choose breast-conservation at the initial diagnosis, there must be a certain willingness to preserve the breast for cosmetic or trauma reasons. Therefore, there are always some patients who desire RBCS after IBTR. However, no prospective studies compared the therapeutic effects of RBCS and SM after IBTR. This meta-analysis was performed to determine the safety of RBCS relative to SM in terms of SLRR, DMR and OS.

The results of previous studies on whether RBCS was better than SM after IBTR varied greatly. Some studies reported that RBCS was not worse than SM (12, 13, 15, 21, 26, 28), and some studies reported the opposite results (8, 10, 11, 14). Other studies asserted that it did not suggest a worse prognosis, although the SLRR of RBCS was higher (25), which is consistent with our conclusions. The SLRR of RBCS was approximately7-31% (8, 13, 14, 29, 30), and the SLRR of SM was approximately 3-32% (8, 13, 21). Nevertheless, these data were based on studies that did not offer secondary radiotherapy and were heterogeneous between groups. Therefore, real-world data must be further refined.

The SLRR of RBCS was initially higher than SM after IBTR of BCS (pRR = 1.87, 95% CI 1.22-2.86, P=0.004). The stratification analysis results showed that the heterogeneity came from the delivery of secondary radiotherapy, which suggests that supplementary radiotherapy after RBCS plays an important role in improving the local control rate. However, there were only two studies that explicitly offered secondary radiotherapy in our study. Previous studies also supported the opinion that secondary radiotherapy after salvage surgery reduces the SLRR (13, 25, 26, 31–38). Su Y et al. (11) reported a worse prognosis after RBCS, poor OS (HR=1.522, 95% CI 1.317-1.759), and poor breast cancer-specific survival (BCSS) (HR =1.666, 95% CI 1.319-2.105). However, for patients with radiotherapy after RBCS, the mortality was comparable to SM regardless of whether radiotherapy was administered after the first breast-conserving surgery, although BCSS was worse for RBCS patients undergoing secondary radiotherapy (HR=1.54, 95% CI 1.037-2.286, p=0.032). Therefore, radiotherapy plays an important role in improving the prognosis after RBCS. However, not all studies agree (25).

Tolerance should also be considered for decisions on secondary radiotherapy after salvage surgery. Some studies reported that a full dose of whole-breast radiotherapy was not tolerated and led to unacceptable toxicity and cosmetic damage. In reality, 75.3% of patients do not receive this radiotherapy (39). More studies showed that supplementary radiotherapy was safe, the side effects were tolerated, and good cosmetic effects were achieved at the same time due to improvements in radiotherapy technology (40–42). Secondary radiation after RBCS may include high-dose external radiation (43), 3-dimensional conformal partial-breast reirradiation (40) and perioperative brachytherapy (26). Hannoun-Levi et al. (31) and Chadha et al. (41) used low-dose-rate multi-catheter implants at centres with considerable brachytherapy experience, and both studies reported excellent outcomes for local control and toxicity. Arthur et al. used external-beam conformal partial-breast radiotherapy (PBI) in RTOG 1014 trial. The Radiation Therapy Oncology Group (RTOG) 1014 trial was the most important prospective study about partial-breast radiotherapy (PBI) after RBCS. One year toxicity report of RTOG 1014 trial was published at a median follow-up of 3.64 years in 2017 (40), there were 4 patients (6.9%) with late grade 3 treatment-related adverse events. Data about effectiveness of treatment and adverse events was updated in 2020 after 5.5 years follow-up (44), four patients (7%) had grade 3 and none had grade 4 adverse events. The 5-year cumulative recurrence of patients who underwent RBCS plus 3D-PBI was 5% (95%CI, 1%-13%) and patients underwent ipsilateral mastectomies was 10% (95%CI, 4%-20%). Both distant metastasis-free survival and overall survival rates were 95% (95%CI, 85%-98%). A study of the GEC-ESTRO Breast Cancer Working Group showed that accelerated PBI with interstitial brachytherapy is feasible and effective in preventing second local recurrence and the OS is at least equivalent to those performed with SM (32). 217 IBTR patients who accepted BCS and whole breast radiation (WBI) were included, the patients were performed with accelerated PBI with interstitial brachytherapy after RBCS, 5 and 10-year SLRR were 5.6% (95% CI: 1.5%–9.5%) and 7.2% (95% CI: 2.1%–12.1%), 5 and 10-year DM were 9.6% (95% CI: 5.7%–15.2%) and 19.1% (95% CI: 7.8%–28.3%), and 5 and 10-year OS were 88.7% (95% CI: 83.1%–94.8%) and 76.4% (95% CI: 66.9%–87.3%). G3-4 complication rate was 11%. Therefore, PBI may be a good re-irradiation method which can provide good therapeutic effectiveness, tolerability and aesthetics after RBCS, which was supported by many other studies (36, 37). To ensure the safety of the second radiotherapy and cosmetic effects, intervention time (perioperative or intraoperative), radiotherapy range (partial or total), and precision (3D conformal) should be considered cautiously.

The results showed no significant difference in the DMR or OS between RBCS and SM after IBTR (DMR: pRR=0.61 (95% CI 0.37-1.01), P=0.05; OS: pRR=0.65 (95% CI 0.39-1.08), P=0.10). No publication bias or differences in sensitivity were observed. Therefore, no significant difference in prognosis was observed in our study despite the lower SLRR of RBCS after IBTR than SM. This conclusion is similar to Sellam Y et al. (25).

An increasing number of recent studies used RBCS (10, 14, 29, 30, 45). The selection of a suitable population for RBCS is very important. The German Society of Radiation Oncology (DEGRO) expert panel guidelines published indications for RBCS in 2016, namely, single disease, size <3 cm, age > 50 years, treatment-free interval (TFI) >48 months, and patient willingness (46). Therefore, some studies suggested that the key factor affecting the prognosis of IBTR is not the method of salvage surgery but the biological behavior of the tumor. IBTR, which has good biological behavior and may be detected early, is suitable for RBCS, but it is not suitable for patients with BRCA mutations (13). It has also been suggested that oestrogen receptor (ER)-positive status and subsequent endocrine therapy should be emphasized (23). From the tumor biological behavior perspective, studies suggested the division of ITBR into two categories: true recurrence (TR) and new primary (NP) (47). The criteria for differentiating TR and NP are location of recurrence, the positive margin of the primary tumor, and pathological characteristics (15). For NP, the DFI was longer, the patients were younger, and the tumor was generally in different quadrants compared to the primary tumor, and these patients had a better survival rate (15, 48). TFI is the most frequently reported key prognostic factor, and it reflects the biological behavior of ITBR (11, 14, 19, 23, 49, 50).

In addition to secondary radiotherapy after salvage surgery, systematic treatment is also important for prognosis. In chemotherapy for isolated locoregional recurrence of breast cancer (CALOR), a study was performed to define the significance of systemic chemotherapy after recurrence for single and operable IBTRs, and the results indicated that the hazard ratio (HR) of the risk of recurrence after salvage surgery between the chemotherapy and no-chemotherapy group was 0.59, and the SM was not a major factor affecting survival (51). Other studies also confirmed the importance of systemic therapy (12, 23).

Our analyses have the following limitations (1). Due to the absence of RCTs, conditions were not balanced between groups, and the SM group had a greater tumor load and later tumor staging (10, 13, 14, 21). Only three studies were basically balanced at baseline (12, 15, 26). However, the SLRR of the RBCS group included in the literature was higher despite the lower tumor load. Therefore, we speculated that the SLRR of RBCS was higher than the real world (2). The time span of the included studies was too long (1993-2019) because the therapeutic effects of breast cancer, especially systemic treatment, have made great progress in recent years, which could cause some deviation.

In conclusion, the SLRR of RBCS is higher for IBTR patients after BCS, but it does not affect survival. Relatively more studies reported that the SLRR was higher after RBCS than after SM, and more studies supported SM. However, due to advancements in radiotherapy technology and systematic treatment, the recurrence rate in the real world may not necessarily be higher. Indications for RBCS must be strictly controlled, namely, tumor size, number of recurrent tumor, lymph node invasion, TFI, age, and biological behavior (such as ER expression, HER2 expression, and BRCA mutation). The importance of secondary radiotherapy and systematic treatment should be emphasized. Methods to avoid the overtreatment of low-risk patients and provide adequate treatment to high-risk patients should be the focus and direction of future research.

## Data Availability Statement

The original contributions presented in the study are included in the article/**Supplementary Material**. Further inquiries can be directed to the corresponding author.

## Author Contributions

CM, WR, and JL: Paper flow Design, Data collection and extraction, Write the manuscript, Analysis of the results. CM and HC: Statistics, Assess the bias and quality. XC: Provide the idea of article, Make important revisions to the paper. All the authors agree the statement above. All authors contributed to the article and approved the submitted version.

## Funding

This work was supported by grants from the Special Fund of Fujian Provincial Department of Finance (2020B010), the Joint Funds for the Innovation of Science and Technology, Fujian Province (2019Y9109), the Young and Middle-aged Teachers Education Scientific Research Project of Fujian Provincial Department of Education (JAT190210), and the Sailing Fund Project of Fujian Medical University (2020QH1016).

## Conflict of Interest

The authors declare that the research was conducted in the absence of any commercial or financial relationships that could be construed as a potential conflict of interest.

## Publisher’s Note

All claims expressed in this article are solely those of the authors and do not necessarily represent those of their affiliated organizations, or those of the publisher, the editors and the reviewers. Any product that may be evaluated in this article, or claim that may be made by its manufacturer, is not guaranteed or endorsed by the publisher.
