# Supplementary material for: Repeat Breast-Conserving Surgery Versus Salvage Mastectomy for Ipsilateral Breast Tumour Recurrence After Breast-Conserving Surgery in Breast Cancer Patients: A Meta-Analysis
Source: Front Oncol. 2021 Nov 23;11:734719. doi: 10.3389/fonc.2021.734719 (PMC8650120; doi:10.3389/fonc.2021.734719)
Supplement: Supplementary file 1 [file DataSheet_1.docx]

**Searching strategy of Pubmed**

#1 "Mastectomy, Segmental"[Mesh]

#2 (partial mastectomy[Title/Abstract]) OR (Mastectomies, Segmental[Title/Abstract]) OR (Segmental Mastectomies[Title/Abstract]) OR (Segmental Mastectomy[Title/Abstract]) OR (Local Excision Mastectomy[Title/Abstract]) OR (Local Excision Mastectomies[Title/Abstract]) OR (Mastectomies, Local Excision[Title/Abstract]) OR (Mastectomy, Local Excision[Title/Abstract]) OR (Segmentectomy[Title/Abstract]) OR (Segmentectomies[Title/Abstract]) OR (Mastectomies, Partial[Title/Abstract]) OR (Mastectomy, Partial[Title/Abstract]) OR (Partial Mastectomies[Title/Abstract]) OR (Limited Resection Mastectomy[Title/Abstract]) OR (Limited Resection Mastectomies[Title/Abstract]) OR (Mastectomies, Limited Resection[Title/Abstract]) OR (Mastectomy, Limited Resection[Title/Abstract]) OR (Lumpectomy[Title/Abstract]) OR (Lumpectomies[Title/Abstract]) OR (Breast-Conserving Surgery[Title/Abstract]) OR (Breast Conserving Surgery[Title/Abstract]) OR (Breast Quadrantectomy[Title/Abstract]) OR (Breast Qu`adrantectomies[Title/Abstract]) OR (Quadrantectomies, Breast[Title/Abstract]) OR (Quadrantectomy, Breast[Title/Abstract]) OR (Surgery, Breast-Conserving[Title/Abstract]) OR (Breast-Conserving Surgeries[Title/Abstract]) OR (Surgeries, Breast-Conserving[Title/Abstract]) OR (Surgery, Breast Conserving[Title/Abstract]) OR (Breast Conservation Therapy[Title/Abstract]) OR (Breast Conservation Therapies[Title/Abstract]) OR (Conservation Therapies, Breast[Title/Abstract]) OR (Conservation Therapy, Breast[Title/Abstract]) OR (Breast-Sparing Surgery[Title/Abstract]) OR (Breast Sparing Surgery[Title/Abstract]) OR (Breast-Sparing Surgeries[Title/Abstract]) OR (Surgeries, Breast-Sparing[Title/Abstract]) OR (Surgery, Breast-Sparing[Title/Abstract]) OR (conservative surgery[Title/Abstract])

#3 #1 OR #2

#4 (Salvage surgery[Title/Abstract]) OR (Salvage surgeries[Title/Abstract]) OR (Salvage operation[Title/Abstract]) OR (Salvage operations[Title/Abstract]) OR (re-operation[Title/Abstract]) OR (Salvage breast conserving surgery[Title/Abstract]) OR (Salvage breast conserving surgeries[Title/Abstract]) OR (repeat breast conserving surgery[Title/Abstract]) OR (repeat breast conserving surgeries[Title/Abstract]) OR (repeat lumpectomy[Title/Abstract]) OR (repeat lumpectomies[Title/Abstract]) OR (salvage lumpectomy[Title/Abstract]) OR (salvage lumpectomies[Title/Abstract]) OR (salvage mastectomy[Title/Abstract])

#5 (Recurrence[Title/Abstract]) OR (Recurrences[Title/Abstract]) OR (Recrudescence[Title/Abstract]) OR (Recrudescences[Title/Abstract]) OR (Relapse[Title/Abstract]) OR (Relapses[Title/Abstract]) OR (ipsilateral breast cancer recurrence[Title/Abstract]) OR (IBTR[Title/Abstract]) OR (ipsilateral breast tumor recurrence[Title/Abstract])

#6 #3 AND #4 AND #5

**Table1 The Newcastle-Ottawa Scale (NOS) results of studies included**

| Author | Year | Representativeness of the exposed cohort | Selection of the non exposed cohort | Ascertainment of exposure | Demonstration that outcome of interest was not present at start of study | Comparability of cohorts on the basis of the design or analysis | Assessment of outcome | | Was follow-up long enough for outcomes to occur | Adequacy of follow up of cohorts | Total score |
| --- | --- | --- | --- | --- | --- | --- | --- | --- | --- | --- | --- |
| Abner,A.L. | 1993 | 1 | 1 | 1 | 1 | 0 | 1 | 1 | | 1 | 7 |
| Alpert,T.E. | 2005 | 1 | 1 | 1 | 1 | 1 | 1 | 1 | | 1 | 8 |
| Chen,S.L. | 2008 | 1 | 1 | 1 | 1 | 0 | 1 | 1 | | 1 | 7 |
| Dalberg,K. | 1998 | 1 | 1 | 1 | 1 | 0 | 1 | 1 | | 1 | 7 |
| Fodor, J. | 2008 | 1 | 1 | 1 | 1 | 0 | 1 | 1 | | 1 | 7 |
| Komoike,Y. | 2005 | 1 | 1 | 1 | 1 | 1 | 1 | 1 | | 1 | 8 |
| Kurtz,J,M | 1988 | 1 | 1 | 1 | 1 | 0 | 1 | 1 | | 1 | 7 |
| Kurtz,J,M | 1990 | 1 | 1 | 1 | 1 | 0 | 1 | 1 | | 1 | 7 |
| Lee.J.H | 2015 | 1 | 1 | 1 | 1 | 0 | 1 | 1 | | 1 | 7 |
| Mccready | 1994 | 1 | 1 | 1 | 1 | 0 | 1 | 1 | | 1 | 7 |
| Salvadori,B | 1999 | 1 | 1 | 1 | 1 | 0 | 1 | 1 | | 1 | 7 |
| Sellam | 2019 | 1 | 1 | 1 | 1 | 0 | 1 | 1 | | 1 | 7 |
| Smanykó,V | 2019 | 1 | 1 | 1 | 1 | 1 | 1 | 1 | | 1 | 8 |
| Voogd,A.C | 1999 | 1 | 1 | 1 | 1 | 0 | 1 | 1 | | 1 | 7 |
| Wapnir,I.L | 2017 | 1 | 1 | 1 | 1 | 1 | 1 | 1 | | 0 | 7 |

Note: The higher score of NOS means lower risk of bias. The total score was≥6 was acceptable.
